# Clinically Relevant ESBL-Producing *K. pneumoniae* ST307 and *E. coli* ST38 in an Urban West African Rat Population

**DOI:** 10.3389/fmicb.2018.00150

**Published:** 2018-02-09

**Authors:** Katharina Schaufler, Kathrin Nowak, Ariane Düx, Torsten Semmler, Laura Villa, Laye Kourouma, Karim Bangoura, Lothar H. Wieler, Fabian H. Leendertz, Sebastian Guenther

**Affiliations:** ^1^Institute of Microbiology and Epizootics, Freie Universität Berlin, Berlin, Germany; ^2^Epidemiology of Highly Pathogenic Microorganisms, Robert Koch Institute, Berlin, Germany; ^3^Microbial Genomics, Robert Koch Institute, Berlin, Germany; ^4^Department of Infectious, Parasitic and Immune-Mediated Diseases, Instituto Superiore di Sanità, Rome, Italy; ^5^Laboratoire Guineo-Allemand, Conakry, Guinea; ^6^Ministère de l'Elevage et des Productions Animales, Conakry, Guinea; ^7^Robert Koch Institute, Berlin, Germany; ^8^Pharmaceutical Biology Institute of Pharmacy, Ernst Moritz Arndt University, Greifswald, Germany

**Keywords:** ESBL, rats, clonal spread, MLST, WGS, one health

## Abstract

High-risk ESBL-producing Enterobacteriaceae (ESBL-E) have been described in wild birds and rodents worldwide. Rats are of special interest not only due to their indicator role for environmental pollution with multi-resistant bacteria but also as possible infection source. Data on the presence of high-risk ESBL-E in urban wildlife from Africa remain scarce, however. Twenty-nine animals from three different rat (*Rattus*) species were captured in the city of Conakry (Guinea, West Africa) in 2015. Rectal swabs were analyzed for ESBL-E using selective media. Species typing and phenotypic antimicrobial resistance analysis to broad-spectrum beta-lactams and other classes of antimicrobials was performed for Enterobacteriaceae-like isolates using the VITEK®2 system (BioMérieux, Germany). Confirmed ESBL-producing *E. coli* and *K. pneumoniae* were whole-genome sequenced and resistance genes, phylogenetic background and genes related to bacterial fitness and virulence were analyzed. In total, six of twenty-nine rats (20%) carried ESBL-E (*K. pneumoniae* and *E. coli*). All ESBL-producers were multi-drug resistant with *bla*_CTX−M−15_ as the dominating ESBL-type. Interestingly, ESBL-associated clonal lineages *E. coli* ST38 and *K. pneumoniae* ST307 were found. The ESBL-plasmid in *K. pneumoniae* ST307 revealed high sequence similarities to pKPN3-307_TypeC, a >200 kbp IncFII plasmid originating from a human clinical ST307 isolate. This was in contrast to the core genome: the rat isolate was distantly related to the human clinical ST307 isolate (27 SNPs/Mbp). In addition, we identified π-fimbrial, capsule 2, and glycogen synthesis clusters in the rodent ST307 isolate, whose involvement in the adaptation to survival outside the host and in human urinary tracts has been suggested. Our results demonstrate the presence of clinically relevant, ESBL-producing *K. pneumoniae* ST307 and *E. coli* ST38 clonal lineages in an urban West African rat population. The human community is likely the initial source of ESBL-E however, rats might function as infection source and transmission hub, accelerated by frequent interactions at a human-wildlife interface.

## Introduction

Extended-spectrum beta-lactamase-producing Enterobacteriaceae (ESBL-E), especially *Klebsiella pneumoniae* and *Escherichia coli*, have been on the rise for years. Diseases these bacteria cause, including diarrhea, septicemia, pneumonia, and urinary tract infections, are not only a cause of child mortality particularly in Africa (Lukac et al., 2015) but are also increasingly difficult to treat due to limitations in antimicrobial therapies (Kanj and Kanafani, 2011).

Besides the threat posed by ESBL-producers, studies report the global spread of *K. pneumoniae* that produce carbapenemases like KPC, often associated with pandemic high-risk clones predominantly of sequence type (ST) 258 and ST307 (Nordmann et al., 2011; Canton et al., 2012; Campos et al., 2016; Lee et al., 2016; Villa et al., 2017).

While research on ESBL-E has mostly focused on human and veterinary clinical contexts, recent findings have shown their prevalence in extra-clinical settings, such as communities, animals, and the environment suggesting a far broader and constant public health threat (Schaufler et al., 2016). Small mammals and rodents (Bonnedahl et al., 2009; Literak et al., 2010) as well as wild birds have been proposed as indicators for the spread of antimicrobial resistance across different environments (Guenther et al., 2010; Allen et al., 2011). While data on urban wild animals as carriers of ESBL-E in Europe, Asia and North-America exist, the role of urban wildlife in Africa remains rather unclear (Skurnik et al., 2006; Literak et al., 2009). We analyzed urban rats captured in the city of Conakry (Guinea, West Africa) for the occurrence of ESBL-E to detect potential reservoirs outside the clinical setting.

## Materials and methods

This study was performed as part of a broader study investigating the role of rodents as potential infection source for various pathogens, in collaboration and under authorization by the Ministry of Livestock and Animal Products (Ministere de l'Elevage et des productions animales) in Conakry, Guinea.

**Figure 1:**
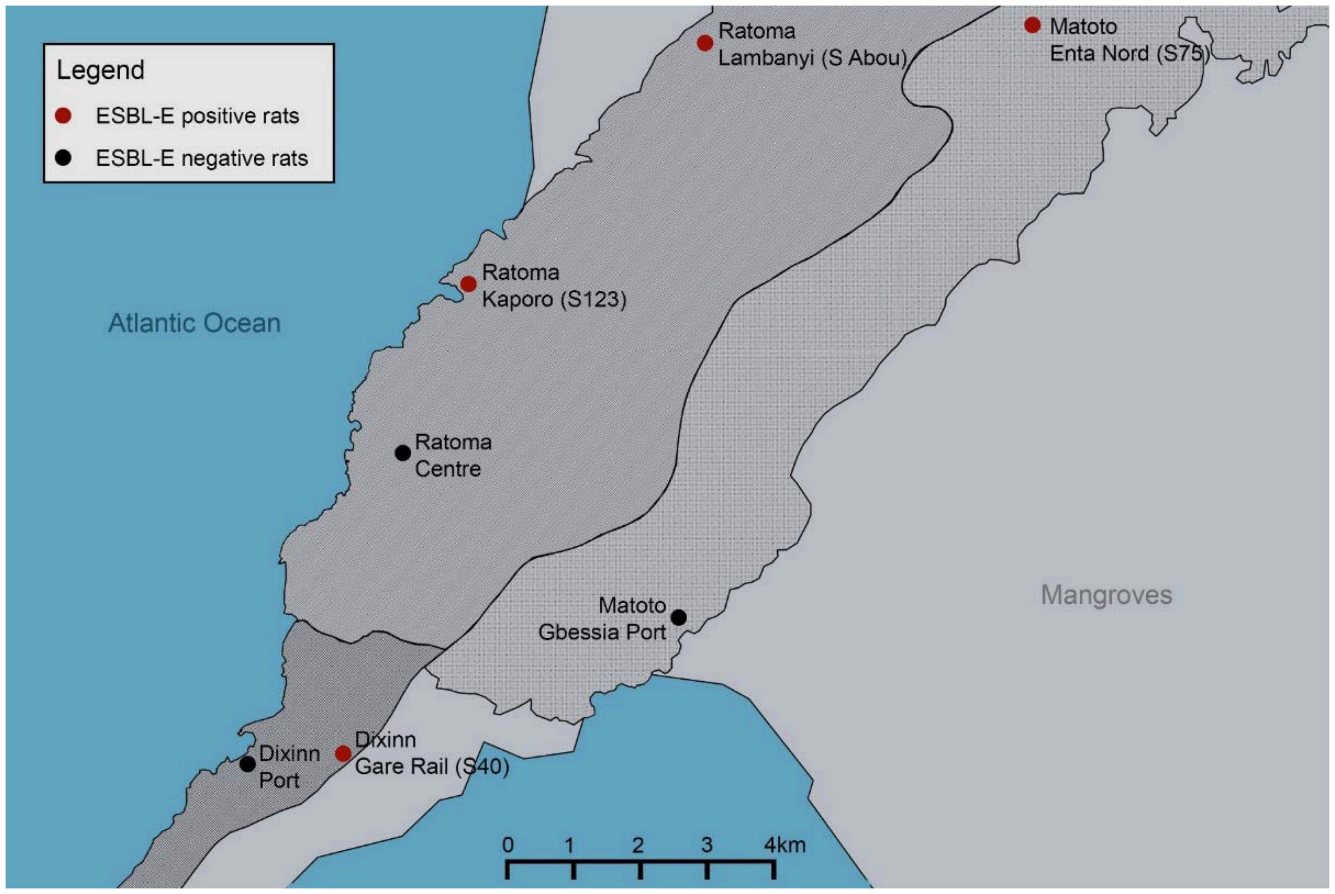
Map of Conakry (Guinea, West Africa) marked in red are ESBL-E-positive rat trapping sites, marked in black are ESBL-E-negative rat trapping sites. Red: S40 (Dixinn Gare Rail: 1 positive out of 9 trapped animals [1/9]), S123 (Ratoma Kaporo: 1/10), S75 (Matoto Enta Nord: 2/4), S Abou (Ratoma Lambanyi: 2/2). Black: Dixinn Port (0/2) Matoto Gbessia Port (0/1) Ratoma Centre (0/1).

Twenty-nine rats from three different species (*Rattus rattus* n = 22, *Rattus norvegicus n* = 6, *Cricetomys gambianus n* = 1) were randomly captured in Conakry (Guinea, West Africa) between November and December 2015. Animals were trapped with life traps (Sherman LFA live trap; H.B. Sherman Traps, Inc., Tallahassee, FL, USA) inside and outside of households in three different, densely populated districts of Conakry (Dixinn [UTM 28P 645031 1055828], Ratoma [UTM 28P 648851 1063078], Matoto [UTM 28P 657469 1066350]; Figure 1). Trapped rodents were handled using appropriate personal protective equipment and were euthanized with a lethal dose of fluorane according to animal welfare guidelines. Each animal was sampled once and rectal swabs (MASTASWAB, Mast Diagnostics Reinfeld, Germany) were shipped to the Institute of Microbiology and Epizootics, Berlin, Germany.

Selection for cefotaxime-resistant and thus ESBL/AmpC-producing *E. coli* and *K. pneumoniae* ssp. *pneumoniae* was carried out by streaking on CHROMagar™ orientation plates (with and without 4 μg/ml cefotaxime; Mast Diagnostica, Reinfeld, Germany). Enterobacteriaceae-like isolates were species-typed by the automated VITEK®2 system (BioMérieux, Germany), which was also used to determine phenotypic antimicrobial resistance to carbapenems and other classes of antimicrobials (card GN38). In addition, isolates growing on CHROMagar^TM^ that contained cefotaxime were confirmed as ESBL-producers using the phenotypic confirmatory test for ESBL-production (CLSI, 2008). Confirmed *E. coli* and *K. pneumoniae* isolates were subjected to whole-genome sequencing (WGS) and plasmid profile analysis as described previously (Schaufler et al., 2016). In brief, WGS was performed using MiSeq Illumina 300 bp paired-end sequencing and a coverage greater than 50 was obtained. After quality control using the NGS tool kit (Patel and Jain, 2012) (70% of bases with a phred score > 20), high quality filtered reads were used for a *de novo* assembly into contiguous sequences (contigs) using Velvet (Zerbino and Birney, 2008). Assembled draft genomes of the isolates were annotated using RAST (Aziz et al., 2008). WGS data was used for genotypic characterization including determination of the multi-locus sequence type (MLST), resistance genes (ResFinder 2.1), and plasmids (PlasmidFinder1.3); all available on the Center for Genomic Epidemiology website (http://www.genomicepidemiology.org).

**Figure 2:**
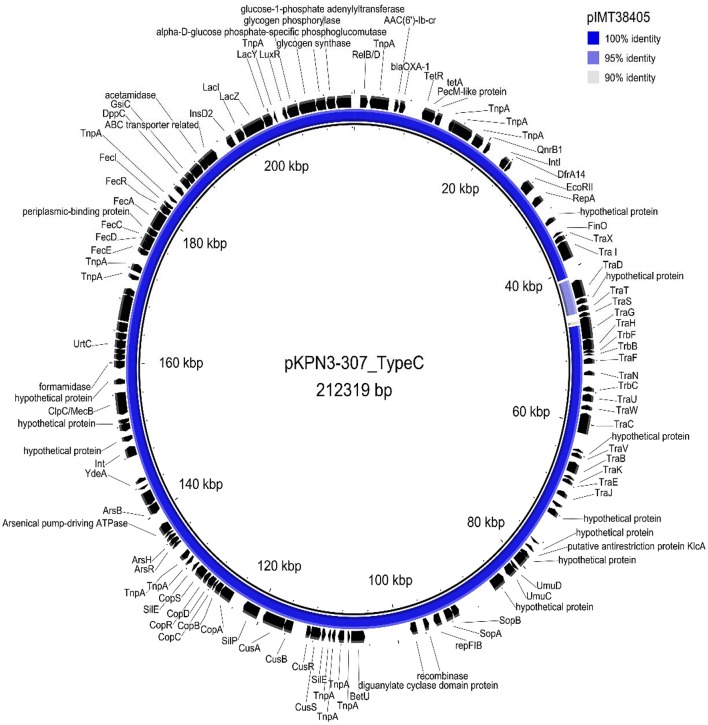
Circular visualization of plasmid pIMT38405 of *K. pneumoniae* ST307 resulting from a mapping of Illumina reads against plasmid pKPN3-307_TypeC (Villa et al., 2017) as a reference sequence using Blast Ring Image Generator (BRIG) (Alikhan et al., 2011).

The plasmid sequence (KY271406) of pKPN3-307_TypeC (Villa et al., 2017) originating from a clinical ST307 isolate from Italy was mapped to the whole-genome of the ST307 isolate IMT38405 using Geneious 6.6. Plasmid contents of both genomes were then compared using Blast Ring Image Generator (BRIG) (Alikhan et al., 2011) (Figure 2).

Genes related to bacterial fitness (π-fimbrial cluster, capsule 2 Enterobacter, glycogen synthesis cluster; (Villa et al., 2017)) were analyzed accordingly. The number of SNPs in the core genome between isolates of the same ST was calculated using Harvest Suite 1.0 (Parsnp) and MEGA 6.0 (http://www.megasoftware.net/).

### Accession numbers

This Whole Genome Shotgun project has been deposited at DDBJ/ENA/GenBank under the Bioproject number PRJNA421654 (PJMH00000000 [IMT38402], PJMI00000000 [IMT38403], PJMJ00000000 [IMT38404], PJMK00000000 [IMT38405], PJML00000000 [IMT38406], PJMM00000000 [IMT38444]).

## Results and discussion

We isolated coliform-appearing bacteria from 93% of all rats (27/29). Twenty percent (6/29) carried ESBL-E (three *K. pneumoniae* [IMT38444, IMT38403, IMT38405] and three *E. coli* [IMT38402, IMT38404, IMT38406]).

All ESBL-producers were multi-drug resistant (Table 1). *Bla*_CTX−M−15_ genes occurred in all strains with the exception of one *bla*_CTX−M−14_- (IMT38444) and one *bla*_CTX−M−9_-carrying strain (*K. pneumoniae* IMT38403). Since *bla*_CTX−M−15_ is the most prevalent ESBL-gene in human samples worldwide (Ewers et al., 2012) it seems likely that humans may act as a reservoir for antimicrobial resistance genes, which can be transmitted to rats and other animals (Gakuya et al., 2001). MLST identified three different STs for *K. pneumoniae* (ST307, ST502, and ST896) and two for *E. coli* (ST38 and ST4684) (Table 1).

**Table 1:** Main characteristics of rat ESBL-producing *K. pneumoniae* and *E. coli*.

**Sample number**	**Bacterial species**	**Sampling point (UTM, city district)**	**Rat species**	**ST**	**Phenotypic resistance**	**Beta-lactam resistance genes**	
**MAIN CHARACTERISTICS**							
IMT38444	*K. pneumoniae*	S 123 (28P 648851 1063078, Ratoma Kaporo)	*R. rattus*	ST-896	CEF, TET, GEN, SXT, TOB	*bla*_CTX−M−14_, *bla*_SHV−11−like_, *bla*_TEM−1B_	
IMT38405	*K. pneumoniae*	S 40 (28P 646660 1055837, Dixinn Gare Rail)	*R. rattus*	ST-307	CEF, TET, ENR, GEN, TOB, SXT	*bla*_CTX−M−15_, *bla*_OXA−1_, *bla*_SHV−28_, *bla*_TEM−1B_	
IMT38403	*K. pneumoniae*	S 75 (28P 657469 1066350, Matoto Enta Nord)	*R. rattus*	ST-502	CEF, CAP, TET, SXT	*bla*_CTX−M−9_, *bla*_DHA−1_, *bla*_SHV−62_	
IMT38402	*E. coli*	S 75 (28P 657469 1066350, Matoto Enta Nord)	*R. norvegicus*	ST-38	CEF, TET, SXT	*bla*_CTX−M−15_, *bla*_TEM−1B_	
IMT38406	*E. coli*	S Abou (n.a., Ratoma Lambanyi)	*R. rattus*	ST-4684	CEF, TET, SXT	*bla*_CTX−M−15_	
IMT38404	*E. coli*	S Abou (n.a., Ratoma Lambanyi)	*R. rattus*	ST-4684	CEF, TET, SXT	*bla*_CTX−M−15_	
**Sample number**	**Aminoglycosides**	**Fluoroquinolones**	**Tetracyclines**	**Chloramphenicol**	**Sulfametoxazole/ Trimethoprim**	**Plasmid content**	**pMLST summary**
**ADDITIONAL GENOTYPIC RESISTANCE AND PLASMIDS**							
IMT38444	*aac(3)-IId-like, strA, strB*	*oqxA-like, oqxB-like, QnrS1*	*tet(A), tet(D)*		*sul1, sul2, dfrA1*	IncFII, IncFIB(K), ColRNAI	IncF[K9:A-:B-]
IMT38405	*aac(3)-IIa-like, strA-like, strB, aac(6')Ib-cr*	*oqxA-like, oqxB-like, QnrB66-like*	*tet(A)*	*catB3-like*	*sul2, dfrA14-like*	IncFIB(K), IncFII(K)	IncF[K7:A-:B-]
IMT38403	*aadA5, strA-like*	*QnrB4*	*tet(B)-like*	*catA1-like*	*sul1, dfrA1, dfrA15*	IncFIA(HI1), IncFIB(K), IncHI1B, IncR, ColRNAI	IncF[F-:A13^*^:B-]
IMT38402	*strA, strB*	*QnrS1*	*tet(A)*	*catB1-like*	*sul2, dfrA14-like*	IncFIB(K), IncFIB(AP001918), ColRNAI	IncF[F-:A-:B53]
IMT38406	*strA-like, strB*	*QnrS1*	*tet(A)-like*		*sul2, dfrA14-like*	ColRNAI	
IMT38404	*strA-like, strB-like*	*QnrS1*	*tet(A)-like*		*sul2, dfrA14-like*	ColRNAI	

*IMT, Institut für Mikrobiologie und Tierseuchen; ST, sequence type; CEF, cefotaxime; CHL, chloramphenicol; ENR, enrofloxacin; GEN, gentamicin; SXT, sulfamethoxazole; TET, tetracycline; TOB, tobramycin. ColRNAI is a small colicin plasmid, n.a. non available*.

Plasmid profiling and bioinformatics analysis revealed the presence of large plasmids (>100 kbp) and incompatibility (Inc) types (e.g., IncFII, IncFIA, IncFIB) often associated with ESBL-plasmids in the *K. pneumoniae* strains (Lee et al., 2016). One *E. coli* strain harbored an IncFIB(K) and IncFIB(AP001918)-type plasmid (ST38, IMT38402, Table 1). Large plasmids, however, were absent in both ST4684 *E. coli* strains (IMT38404 and IMT38406) suggesting a chromosomal ESBL-gene location. This was supported by WGS results of both *E. coli* strains as (i) the *bla*_CTX−M_ genes were detected on very large contigs (>250 kbp), (ii) BLAST analysis revealed mostly chromosomal hits for the CTX-M-carrying contigs, and (iii) no ESBL-associated Inc-types were identified. The mobile element Tn3 was found 300 bp downstream of CTX-M-15 and ISEc9 (ISEcp1-like element) directly upstream in an otherwise chromosomal background, which reinforced our hypothesis.

The chromosomal integration of ESBL-genes in *E. coli* from wild birds has previously been reported by Guenther et al. (2017). However, in this previous work, ST38 carried chromosomally-encoded ESBLs, contrarily to our current findings. The comparison of these wild bird ST38 to our rat ST38 isolates revealed a high number of SNPs (>1800 SNPs/Mbp) demonstrating their phylogenetically distinct character. *E. coli* ST38 is a prevalent human clinical pathogen but has also been reported on samples from wildlife as mentioned above (Turton et al., 2016).

Interestingly, *K. pneumoniae* ST307 has emerged worldwide in different locations and has been described as a putative high-risk clone associated with the production of carbapenemase KPC-2 and/or CTX-M-15 (Zhang et al., 2016; Kim et al., 2017). It has been frequently reported in human clinical samples from Italy, Korea, Pakistan, and Morocco and in pets from Japan. Although in small numbers detected, some of the rat strains resemble common pathogenic STs suggesting spill-over of clinical strains into an urban rat population in West Africa or vice versa.

Interestingly, comparison of the *K. pneumoniae* ST307 (IMT38405) ESBL-plasmid (~210 kbp) with a recently published *K. pneumoniae* WGS of an isolate with the same ST (Kim et al., 2017; Villa et al., 2017) isolated from a human clinical sample demonstrated high plasmid similarities (pKPN3-307_TypeC, KY271406)(Figure 2). Approximately 180 kbp of the rat isolate's plasmid sequence was 100% identical to the 212 kbp reference plasmid KPN3-307_TypeC. The remaining 30 kbp showed a similarity of 95%. Differences were only observed for one IS6 insertion family element (IS6100) upstream of the *tra* region. SNPs were found in *finO* and several genes from the *tra* operon.

When comparing the whole-genome sequence of IMT38405 to three recently published *K. pneumoniae* ST307 (Civ4, KH43, KL49; Villa et al., 2017), we detected between 155 and 432 SNPs in total (27–78 SNP/Mbp), excluding a recent clonal spread. Considering the SNPs Mbp^−1^ ratio of clonal EHEC strains during the outbreak (1.8 SNPs/Mbp) in Germany in 2011, our SNPs Mbp^−1^ ratio is tenfold higher, which indicates several years of separate evolution (Grad et al., 2012; de Been et al., 2014).

However, the occurrence of almost identical plasmids underlines the importance of plasmid-driven spread and transmission of antimicrobial resistances independent of the host's core genome.

We identified *K. pneumoniae* ST307 chromosomally-encoded genes, whose involvement in the adaptation to survival outside the host and in the human urinary tract has been previously suggested: the π-fimbrial cluster enabling bacterial mobility, and a serum resistance-conferring capsule 2 (Villa et al., 2017). A plasmid-encoded glycogen synthesis cluster (Villa et al., 2017), which may contribute to environmental survival through energy advantages and thus possibly to the bacterial spread between different hosts and settings, was also present. Carriage of these genes might be involved in the success of KPC-2 producing *K. pneumoniae* ST307 but probably predates the uptake of carbapenemase-plasmids as we found the same genes in our CTX-M-15-producing isolates.

In summary, our results demonstrate the presence of clinically relevant, ESBL-producing *K. pneumoniae* and *E. coli* clones in an urban West African rat population. Given the sanitary conditions in this region, rats could act either as indicators or environmental reservoirs of ESBL-E with a risk of transmission to humans with a putative public health impact. Clearly, more in-depth data are needed to define the risk of ESBL-E transmission by rats.

## Author contributions

SG, KS, FL, and KN conceived and designed the experiments; KN, FL, AD, LK, LV, and KB collected the data and samples; KS performed laboratory analysis; KS, SG, LW, and TS analyzed the data; KS and SG wrote the article. All authors have read and approved the final draft of the manuscript.

### Conflict of interest statement

The authors declare that the research was conducted in the absence of any commercial or financial relationships that could be construed as a potential conflict of interest.
